# Host-related Determinants of Response to Immunotherapy in Non-small Cell Lung Cancer: The Interplay of Body Composition, Metabolism, Sex and Immune Regulation

**DOI:** 10.1007/s11912-025-01718-7

**Published:** 2025-12-23

**Authors:** Valentina Santo, Leonardo Brunetti, Federica Pecci, Marianna Peroni, Giulia Barnini, Francesco Paoloni, Sebastiano Buti, Marcello Tiseo, Biagio Ricciuti, David James Pinato, Alessio Cortellini

**Affiliations:** 1 Operative Research Unit of Medical Oncology, Fondazione Policlinico Universitario Campus Bio-Medico, Via Alvaro del Portillo 200, Roma, 00128 Italy; 2 Department of Medicine and Surgery, Università Campus Bio-Medico di Roma, Via Alvaro del Portillo 200, Roma, 00128 Italy; 3 Lowe Center for Thoracic Oncology, Dana-Farber Cancer Institute, Boston, MA USA; 4 Department of Surgery and Cancer, Imperial College of London, Hammersmith Hospital Campus, Du Cane Road, London, UK; 5 Department of Medicine and Surgery, University of Parma, Parma, Italy; 6 Medical Oncology Unit, University Hospital of Parma, Parma, Italy; 7 Department of Translational Medicine (DIMET), University of Piemonte Orientale “A. Avogadro”, Novara, Italy

**Keywords:** NSCLC, Metabolism, Host-factors, Diabetes, Lipid metabolism, Body composition

## Abstract

**Purpose of Review:**

Non-small cell lung cancer (NSCLC) is a biologically and clinically heterogeneous disease. In addition to tumor-intrinsic characteristics, clinical outcomes from immune checkpoint inhibitors (ICIs) are influenced by a variety of host-related factors. This review aims to summarize current evidence on how body composition, metabolic comorbidities, sex, and systemic inflammation shape anti-tumor immunity and affect immunotherapy efficacy.

**Recent Findings:**

Emerging data suggest that altered body composition, including obesity and sarcopenia, may modulate ICI outcomes, giving rise to the so-called “obesity paradox”, which appears inconsistent across tumor types and may reflect disease-specific nutritional and immunological profiles. Likewise, metabolic disorders such as type 2 diabetes and dyslipidemia can promote chronic inflammation and immune exhaustion, potentially dampening ICI activity. Advances in cross-sectional imaging and molecular profiling are refining the characterization of host–tumor–immune interactions and providing novel predictive insights.

**Summary:**

Host-related determinants play an integral role in shaping response to ICIs in NSCLC. A deeper understanding of the dynamic continuum linking metabolism, body composition, systemic inflammation, and immune regulation may enable more precise patient stratification and open opportunities for personalized immunotherapy strategies.

## Introduction

The clinical efficacy of immune checkpoint inhibitors (ICIs) is shaped not only by tumor-intrinsic features but also by a complex network of host-related factors. Elements such as nutritional status, metabolic comorbidities and systemic inflammation significantly modulate the antitumor immune response [1]. These parameters influence both the pharmacodynamics of ICIs [2, 3] and the functional competence of immune effector cells, ultimately affecting treatment outcomes. Understanding how host characteristics interact with immune function and tumor biology is essential for optimizing patient selection and tailoring therapeutic strategies in the immunotherapy era.

**Fig. 1:**
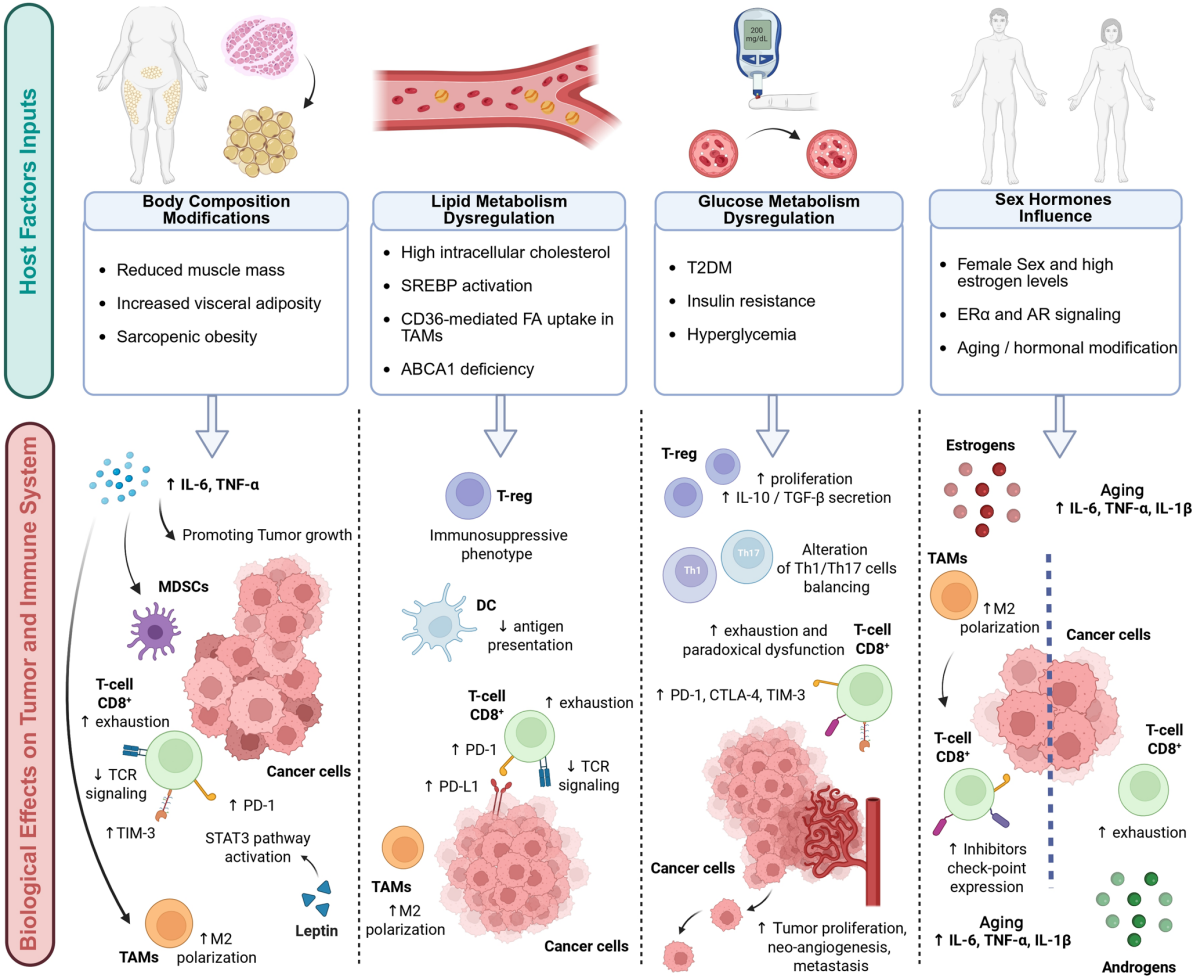
Graphical abstract: Schematic overview of key host-related metabolic and hormonal factors influencing immune response and tumor progression in NSCLC. Body composition alterations (e.g., sarcopenic obesity), glucose metabolism dysregulation (e.g., T2DM, hyperglycemia), lipid metabolism alterations (e.g., SREBP activation, CD36-mediated fatty acid uptake), and sex hormone signalling (e.g., ERα and AR) modulate both immune cell function and tumor behavior. These host factors influence inflammation, immune suppression, and tumor progression through distinct but interconnected mechanisms. TAMs: tumor-associated macrophages; FA: fatty acids; ERα: estrogen receptor alpha; AR: androgen receptor. Figure created with Biorender.com

As summarized in the graphical abstract (Fig. 1), this review explores the major host-related factors (metabolic, hormonal, and immunologic) that shape tumor–immune interactions and potentially affect response to immunotherapy in non-small cell lung cancer (NSCLC).

For this narrative review we searched PubMed and Embase for articles published from January 2010 to July 2025 using combinations of terms including ‘non-small cell lung cancer’, ‘immunotherapy’, ‘checkpoint inhibitor’, ‘body composition’, ‘sarcopenia’, ‘obesity’, ‘lipid’, ‘cholesterol’, ‘diabetes’, ‘hyperglycemia’, ‘sex’, ‘estrogen’, ‘androgen’. We prioritized peer reviewed studies in adults, with a focus on immunotherapy treated cohorts and translational studies relevant to mechanism. References were complemented by back citation searches.

## Body Composition and Obesity

Body composition (BC), including both muscle mass and adipose tissue is closely linked to a patient’s overall health status and the presence of comorbidities. Alterations in BC not only reflect underlying metabolic conditions such as obesity, sarcopenia or cachexia, but also influence immune competence, treatment tolerance and therefore clinical outcomes across a wide range of diseases, including cancer [4].

Emerging evidence highlights the connection between BC and immunity, suggesting that changes in adipose and muscle tissue can modulate key immunoregulatory pathways, impacting T cell function, systemic inflammation and the tumor microenvironment (TME) [5–8]. Given these complexities, a deeper evaluation of the interplay between BC and immune response is essential to identify novel biomarkers beyond conventional metrics, enabling more accurate predictions of immunotherapy outcomes across all cancers including NSCLC.

### Obesity in Lung Cancer Development and Outcomes

Obesity is estimated to account for 4–8% of all cancer cases and is associated not only with increased cancer risk but also with worse outcomes among cancer survivors, including higher recurrence and mortality rates [9].

This biological connection between obesity and cancer is complex and multifactorial. Adipose tissue (AT) acts as an active endocrine organ, secreting enzymes and signalling molecules that influence tumorigenesis. For instance, aromatase-mediated conversion of androgens to estrogens in AT contributes to the development of different tumors such as breast, endometrial, and ovarian cancers [10, 11]. Additionally, hyperinsulinemia, common in obese patients, enhances tumor growth through the activation of the insulin–IGF-1 axis, particularly in colorectal, renal, prostate, and endometrial cancers [12].

Nevertheless, historical epidemiological data report an inverse association between the body mass index (BMI) and lung cancer risk, likely due to residual confounding influence of smoking [10]. Even though no final evidence supports a direct protective effect of adiposity in this setting, a recent mendelian randomization analysis, adjusted for smoking behaviours, confirmed a direct inverse association between increasing BMI and lung adenocarcinoma risk and with positive causal effect on the development of small cell lung cancer, whereas no significant direct effect was observed for squamous cell carcinoma [13].

Obesity also induces a chronic pro-inflammatory state driven by altered adipokine secretion, specifically increased leptin and decreased adiponectin, which promotes oxidative stress, DNA damage, and the activation of carcinogenic pathways, collectively promoting tumor progression [14, 15].

However, a large meta-analysis involving over 6 million patients with cancer found that while obesity was generally associated with increased overall and cancer-specific mortality, exceptions existed in patients with lung cancer, renal cell carcinoma (RCC), and melanoma, where obesity correlated with improved survival, supporting the hypothesis of a protective role for obesity in selected tumor types [9], that appears to be particularly relevant in the context of cancer immunotherapy.

Recent studies have reported better survival outcomes in patients with cancer with a BMI ≥ 25 kg/m² compared to those with normal or low BMI, suggesting a potential protective effect in obese patients, a phenomenon known as the “obesity paradox“ [16, 17].

### Obesity-induced Immune Remodelling: from Dysfunction to Therapeutic Vulnerability

AT, including both white and visceral compartments, exerts immunoregulatory functions, hosting a distinct immune cell population, including macrophages, invariant natural killer T cells, and regulatory T cells (Tregs), whose imbalance contributes to obesity-related inflammation and metabolic dysregulation [5].

Among these immune cells infiltrating AT, macrophages are the most abundant and serve as key mediators of obesity-induced inflammation through the secretion of pro-inflammatory cytokines, such as interleukin-6 (IL-6) and tumor necrosis factor- α (TNF-α) [18].

Within the TME, obesity has been shown to promote a shift toward an M2-like macrophage phenotype [19], which is immunosuppressive and associated with impaired anti-tumor immunity and tumor growth [20]. Myeloid-derived suppressor cells (MDSCs), expanded in obesity in response to elevated levels of IL-6 and other cytokines, contribute to this immune evasion by suppressing CD8^+^ T cell activity and further promoting M2 polarization [21].

Obesity impairs T cell functionality by reducing T cell receptor (TCR) diversity and sensitivity and promoting T cell exhaustion [18]. While CD8^+^ T cells are among the most affected, Tregs are also modulated by obesity. Wang et al. report that obesity and type 2 diabetes are associated with a reduction in Tregs, contributing to a Th17/Treg imbalance that promotes chronic inflammation and insulin resistance [22]. Interestingly, Ringel et al. recently demonstrated that obesity-induced metabolic remodelling within the TME promotes tumor growth by impairing CD8^+^ T cell function, with tumor and immune cells displaying distinct metabolic adaptations to obesity [23].

Taken together, obesity promotes a TME characterised by chronic low-grade inflammation and immunosuppressive features, which together impair effective anti-tumor immunity and facilitate immune escape.

On the other hand, consistent evidence suggested that obesity-related immune dysregulations may render patients more susceptible to immune checkpoint inhibition: by targeting pathways activated in the context of chronic inflammation and T cell exhaustion, ICIs can partially reverse obesity-induced immune dysfunction.

In diet-induced obese mice, immune dysfunction progressively develops with increasing weight, with enhanced PD-1 expression and functional exhaustion of both CD4^+^ and CD8^+^ T cells, especially in peripheral blood, liver, and spleen. These T cells exhibit impaired proliferation and reduced production of key effector cytokines such as interferon- γ (IFN-γ) and TNF-α [24].

In tumor-bearing models, obese mice show more aggressive tumor growth and a TME enriched in dysfunctional CD8^+^ tumor-infiltrating lymphocytes expressing high levels of PD-1, Tim3, and Lag3. Transcriptomic analyses of CD8^+^ T cells from these mice reveal upregulation of exhaustion-, senescence-, and transforming growth factor-β (TGF-β)–related gene signatures, along with metabolic reprogramming [24].

A key mechanistic link is represented by leptin, that enhances PD-1 expression on CD8^+^ T cells via *STAT3* signalling, contributing to their dysfunctional phenotype. In leptin-deficient (ob/ob) or leptin receptor–deficient (db/db) mice, PD-1 expression is reduced, and T cells retain better proliferative capacity, highlighting leptin as a driver of obesity-associated T cell exhaustion [24].

Interestingly, despite this immunosuppressive phenotype, diet-induced obesity mice demonstrate improved responses to anti–PD-1 therapy, with greater tumor regression and enhanced T cell effector function, a paradox observed across multiple tumor models including melanoma and lung carcinoma [24, 25].

Further evidence suggests that obesity-related immune dysfunction impairs tumor immunosurveillance, increasing cancer risk. However, tumors arising in this context may undergo less stringent immunoediting, remaining more immunogenic and thus more responsive to immune checkpoint blockade [26].

These findings collectively support a model where obesity fosters an immunosuppressive TME, yet simultaneously primes the tumor for enhanced sensitivity to PD-1/PD-L1 blockade.

### Clinical Evidence on the Obesity Paradox in Patients with NSCLC Treated with ICI

Several studies suggest that patients with a BMI >25 kg/m² experience better outcomes with ICIs compared to those with normal BMI [27]. In particular, in patients with NSCLC, a higher BMI appears to be associated with improved survival following ICI treatment [28–33].

A pooled analysis of four clinical trials including 2,110 patients with advanced NSCLC, revealed that a higher BMI was associated with improved overall survival (OS) in patients treated with atezolizumab with no effect among the control cohort of patients treated with docetaxel. Specifically, overweight (BMI 25–29.9 kg/m²) and obese patients (BMI ≥ 30 kg/m²) had significantly longer OS compared to those with normal BMI, with the strongest effect observed in the obese group. Moreover, when overweight and obese patients were grouped together, they also experienced a modest but statistically significant improvement in progression free survival (PFS). These associations appeared stronger in patients with PD-L1–positive tumors [28].

In a multicenter retrospective study, baseline obesity (BMI ≥ 30 kg/m²) was significantly associated with improved clinical outcomes in patients with metastatic NSCLC with PD-L1 ≥ 50% receiving first-line pembrolizumab. Obese patients had a higher objective response rate (ORR), longer PFS, and longer OS, compared to normal-weight patients. In contrast, no association was observed in the control cohort of patients treated with standard first line platinum-based chemotherapy at the same centers. Moreover, a BMI gain of ≥ 1.4% during treatment was independently associated with improved ORR, PFS, and OS in both cohorts, with a stronger effect seen in the pembrolizumab group [29].

Additional evidence, including recent meta-analyses and large multicenter cohorts, has further investigated the obesity paradox in the context of immunotherapy for NSCLC, though results remain inconsistent. A meta-analysis by Zhang et al. [34], which included 4,602 patients with NSCLC treated with ICIs, reported that overweight and obese patients achieved significantly longer PFS and OS compared to normal-weight individuals, supporting a possible survival benefit in this population. However, findings from real-world data and large multicenter studies challenge the robustness of this association. In a cohort of over 7,000 patients, Nie et al. [35] found no impact of BMI on survival outcomes in those receiving either immunotherapy or chemoimmunotherapy, and only observed a survival advantage among obese male patients treated with chemotherapy. Similarly, Cortellini et al. [36] showed no independent prognostic effect of BMI in patients treated with first-line chemoimmunotherapy combinations, while results from a large Japanese registry analysis by Ihara et al. [37] further confirmed the absence of a protective effect of obesity in patients receiving ICIs, with survival benefits limited to those with BMI < 28 kg/m².

Table 1 summarises key studies evaluating the impact of BMI on immunotherapy outcomes in patients with advanced NSCLC.

**Table 1:** Key studies evaluating the impact of body mass index (BMI) on immunotherapy outcomes in advanced non-small cell lung cancer (NSCLC)

Study	Study Design	Patient Population	Treatment type	BMI Categories	PFS	OS	ORR
Kichenadasse et al., 2020 [28]	Pooled analysis of 4 clinical trials (OAK, POPLAR, BIRCH, FIR)	2,110 patients	Atezolizumab (vs. docetaxel) as first or subsequent line	Obese (BMI ≥ 30 kg/m^2^), overweight (BMI 25–29.9 kg/m^2^), normal weight (BMI 18.5–24.9 kg/m^2^)	No significant difference in overall population; in PD-L1 positive HR 0.78, *p* = 0.04 in obese vs. normal weight	HR 0.64, *p* < 0.001 in obese vs. normal weight; no benefit in PD-L1 negative	NA
Cortellini et al., 2020 [29]	Real-world multicenter retrospective cohort	962 patients with PD-L1 TPS ≥ 50% (control cohort of 426 patients)	Pembrolizumab (vs. platinum-based CHT) as first line	Obese (BMI ≥ 30 kg/m^2^), overweight (BMI 25–29.9 kg/m^2^), normal weight (BMI 18.5–24.9 kg/m^2^)	aHR = 0.61, *p* = 0.0012 in obese vs. normal weight	aHR = 0.70, *p* = 0.0474 in obese vs. normal weight	57.5% obese vs. 43% normal weight, *p* = 0.007
Ying et al., 2022 [31]	Retrospective study	212 patients	ICI	Higher BMI (BMI ≥ 25 kg/m^2^), lower BMI (BMI < 25 kg/m^2^)	8.1 months in higher BMI vs. 5.3 months in lower BMI, *p* = 0.0034	10.8 months in higher BMI vs. 5.9 months in lower BMI, *p* = 0.0008	NA
Li et al., 2022 [33]	Retrospective cohort study	459 patients	ICI	Obese (BMI ≥ 40 kg/m^2^), normal BMI (18.5–25 kg/m^2^)	NA	HR = 0.46, *p* = 0.032 in obese vs. normal BMI	NA
Lee et al., 2023 [32]	Retrospective cohort study	820 patients	ICI	Obese (BMI ≥ 25 kg/m^2^), overweight (BMI 23–24.9 kg/m^2^), normal weight (BMI 18.5–22.9 kg/m^2^)	aHR = 0.75, *p* = 0.005 in obese vs. Normal weight	aHR = 0.64, *p* < 0.001 in obese vs. normal weight	NA
Osataphan et al., 2023 [30]	Retrospective cohort study	502 patients	ICI +/- CHT	Obese (BMI > 30 kg/m^2^)	NA	aHR 0.70, *p* = 0.042 in obese vs. non-obese	NA
Palmer et al., 2021 [38]	Retrospective cohort study	178 patients	ICI +/- CHT	Obese (BMI ≥ 30 kg/m^2^), overweight (BMI 25–29.9 kg/m^2^)	7.4 months obese/overweight vs. 8.1 months normal weight, *p* = 0.2	15.9 months obese/overweight vs. 16.8 months normal weight, *p* = 0.5	37% obese/overweight vs. 52% (normal weight), *p* = 0.06
Cortellini et al., 2022 [36]	Multicenter retrospective cohort	853 patients	ICI + CHT	Obese (BMI ≥ 30 kg/m^2^), overweight (25 ≤ BMI ≤ 29.9 kg/m^2^), normal weight (18.5–24.9 kg/m^2^), underweight (< 18.5 kg/m^2^)	aHR = 1.04, *p* = 0.72 in obese vs. normal weight	aHR = 0.99, *p* = 0.96 in obese vs. normal weight	NA
Antoun et al., 2022 [39]	Secondary analysis of a prospective cohort	389 patients	ICI	Overweight/ Obese (BMI ≥ 25 kg/m^2^), normal weight (18.5–24.9 kg/m^2^)	NA	aHR = 0.80, *p* = 0.13 in obese vs. normal weight	NA
Ihara et al., 2024 [37]	Retrospective cohort study	31,257 patients	ICI vs. conventional CHT	Obese (BMI ≥ 30 kg/m^2^), overweight (25–29.9 kg/m^2),^ normal weight (18.5–24.9 kg/m^2^)	NA	Obese/overweight with lower risk of mortality (cut-off BMI 28 kg/m^2^); no treatment specific differences for OS	NA
Nie et al., 2024[35]	Multicenter retrospective cohort	7,021 patients (multiple cohorts)	CHT vs. CHT-ICI vs. ICI	CGDB, CHOWELL-IMMU cohorts: WHO definition; other cohort: Asia Pacific WHO definition	No impact of BMI on PFS in patients receiving ICI or CHT	No impact of BMI on OS in patients receiving ICI or CHT	No differences in ORR

*BMI* body mass index; *ICI* immune checkpoint inhibitors; *CHT c*hemotherapy; *PFS* progression free survival; *OS* overall survival; *ORR* objective response rate; *HR* hazard ratio; *p p*-value

Altogether, while several studies support that higher BMI may be associated with improved survival in patients with NSCLC treated with ICIs, the evidence remains inconclusive and likely confounded by factors such as treatment selection, sex, performance status, physical activity, systemic inflammation and BC.

As highlighted by Antoun et al. [39], the association between obesity and improved outcomes disappears when adjusting for cancer-associated weight loss and reduced skeletal muscle mass, emphasizing the importance of cachexia and metabolic reserve in this disease setting. These findings suggest that the survival benefit observed in overweight patients with NSCLC may be partly driven by broader nutritional and functional factors, rather than reflecting a purely protective effect of adiposity itself. Nevertheless, a potential immunomodulatory role of excess adipose tissue cannot be excluded. Future studies should incorporate more refined and dynamic metrics of body composition to better elucidate the complex interplay between host metabolism, tumor biology, and immune responsiveness.

Beyond muscle loss and systemic inflammation, neuroendocrine signals that track energetic stress may shape tumour behaviour in lung cancer. Low body mass index is associated with activation of the ghrelin growth hormone secretagogue receptor pathway and increased neuronal secretion of neuropeptide Y, which in preclinical and translational studies promoted brain colonisation through NPY Y5 receptor signalling and lipogenic reprogramming [40]. In this model, reversing the low body mass index phenotype or blocking Y5 receptor signalling suppressed brain metastasis. These data support the concept that catabolic states can rewire tumour and immune metabolism through brain cancer crosstalk. Although not yet linked directly to immunotherapy response, they reinforce the rationale to prevent or treat cancer associated anorexia and weight loss. Ghrelin agonists are under clinical evaluation for appetite and weight endpoints [41] and could be explored as supportive care in patients with cancer receiving immunotherapy, while recognising that randomised evidence on immunotherapy outcomes is currently lacking.

### Beyond BMI: Alternative Metrics for Immunotherapy Response

Recently, research has been focusing on finding more accurate alternative tools to BMI in order to explain the obesity paradox. Despite being easy to measure, BMI might not always represent a reliable surrogate of BC as it does not take into consideration the correct proportion and distribution of adipose and muscle mass [42]. The protective effect of obesity is strictly dependent on both body composition and on systemic inflammation status, and different BC phenotypes might be related to specific patient’s immunology and consequently influence immunotherapy response [43, 44].

To overcome BMI limitations and describe thoroughly the BC, cross-sectional imaging techniques (i.e. computed tomography [CT] or fat-referenced quantitative magnetic resonance imaging [MRI]) are being increasingly used to accurately quantify and distinguish abdominal adipose tissue distribution (visceral vs. subcutaneous) and muscle compartments in BC studies [45, 46]. Imaging-derived BC features have been usually assessed through a two-dimensional (2D) method, using a segmentation at the L3 abdominal level, although current evidence suggest that three-dimensional (3D) measurements, using multiple segmentation, represent a preferable technique [47, 48].

Several studies have investigated the association between baseline CT-derived adiposity parameters and clinical outcomes in cancer patients treated with ICIs. A recent systematic review and meta-analysis by Lou et al. [45]. showed that higher values of subcutaneous and visceral fat, quantified as subcutaneous fat area (SFA), subcutaneous fat index (SFI), and visceral fat index (VFI), were generally associated with improved overall and progression-free survival. However, these associations were not uniform across all fat compartments: while subcutaneous and visceral fat consistently correlated with better outcomes, intermuscular fat, which reflects ectopic fat infiltration between muscle fibers, showed no prognostic value. This likely reflects the differing biological functions of each depot, only certain AT may modulate systemic inflammation and immunity in ways relevant to cancer progression and treatment response.

Retrospective evidence investigating the role of BC parameters as predictors of ICIs efficacy are summarised in Table 2.

**Table 2:** Retrospective evidence on body composition (BC) parameters as predictors of immune checkpoint inhibitors (ICIs) efficacy

Trial	Patients receiving ICIs *n*.	Type of cancer	Parameters	PFS (months)	OS (months)	ORR or DCR or CB
Young et al., 2020 [49]	287	Melanoma	SMG (High vs. Low)	HR 1.21 (95% CI 0.70–2.10) *p* = 0.48	HR 0.99 (95% CI 0.53–1.83) *p* = 0.97	OR: 0.90 (95% CI 0.36–2.28) *p* = 0.83
			TATI (High vs. Low)	HR 1.71 (95% CI 1.01–2.87) *p* = 0.04	HR 1.44 (95% CI 0.80–2.61) *p* = 0.22	OR: 0.39 (95% CI 0.15–1.02) *p* = 0.06
Trestini et al., 2024 [50]	134	NSCLC	Sarcopenia (Yes vs. No)	HR 2.24 (95% CI 1.37–3.67) *p* = 0.001	HR 4.68 (95% CI 2.44–8.99) *p* < 0.0001	OR: 5.56 (95% CI 2.46–12.6) *p* < 0.0001
			IMAT (as continuous variable)	HR 2.26 (95% CI 1.40–3.63) *p* = 0.002	HR 3.18 (95% CI 1.72–5.88) *p* < 0.0001	OR: 1.83 (95% CI 1.22–2.83) *p* = 0.0001
McManus et al., 2023 [51]	99	RCC	SMI (High vs. Low)	HR 2.433 (95% CI, 1.397–4.238) *p* = 0.0017	27.01 vs. 42.74 months HR 1.728 (95% CI, 0.909–3.285) *p* = 0.0952	NA
			SATI (High vs. Low)	HR 1.641 (95% CI, 1.023–2.632) *p* = 0.0398	NA	NA
Makrakis et al., 2023 [52]	52	NSCLC	LSMI (Low vs. High)	3.30 vs. 7.33, *p* = 0.040	6.37 vs. NR, *p* = 0.009	NA
			SFI (Low vs. High)	2.97 vs. 5.77, *p* = 0.135	5.43 vs. 14.03, *p* = 0.020	
Wang et al., 2022 [53]	251	RCC	SAT% (as continuous variable)	HR 0.02 (CI 95% CI, 0.01–0.11)	HR 0.05 (95% CI, 0.01–0.39)	NA
Takenaka et al., 2022 [54]	114	HNSCC	SMI (Low vs. High)	HR 1.74 (95% CI, 1.04–2.92)	HR 2.06 (95% CI, 1.16–3.67)	DCR: OR 0.39 (95% CI, 0.15–0.97)
			VAI (Low vs. High)	HR 2.07 (95% CI, 1.15–3.73)	NA	ORR: OR 0.38 (95% CI, 0.15–0.94)
Lee et al., 2022 [55]	266	Melanoma	VFI (High vs. Low)	NA	49.1 vs. 38.0, *p* < 0.001	DCR 69.6% vs. 56.5%, *p* = 0.028
						ORR 38% vs. 41.8%, *p* = 0.535

*BMI* body-mass index; *CI* confidence interval; *HCC* hepatocellular carcinoma; *HNSCC* head and neck squamous cell carcinoma; *HR* hazard ratio; *ICIs* immune checkpoint inhibitors; *IMAT* intermuscular adipose tissue area; *LSMI* lumbar skeletal muscle index; *NA* not available; *NR* not reached; *NSCLC* non-small cell lung cancer; *OR* odd ratio; *ORR* objective response rate; *OS* overall survival; *PFS* progression-free survival; *RCC* renal cell carcinoma; *SATI* subcutaneous adipose tissue index; *SAT%* subcutaneous adipose percentage; *SFI* subcutaneous fat index; *SMA* skeletal muscle area; *SMI* skeletal muscle index; *SMD* skeletal muscle density; *SMG* skeletal muscle gauge; *TATI* total adipose tissue index; *VAI* visceral adiposity index; *VFI* visceral fat index

In addition to adiposity, skeletal muscle mass has emerged as a key body composition parameter with significant prognostic and immunological implications. CT imaging, already employed to assess fat distribution, also enables accurate quantification of muscle compartments allowing for the identification of patients with low muscle mass (sarcopenia). These measurements, such as skeletal muscle area (SMA), skeletal muscle index (SMI), and muscle density, are increasingly recognized as critical markers of systemic metabolic reserve and immune competence [56].

In patients with NSCLC, low skeletal muscle mass has been associated with worse clinical outcomes and may negatively influence the response to ICIs [57, 58]. This is especially relevant in the context of sarcopenic obesity (SO), a condition defined by the coexistence of excess adiposity and reduced muscle mass. SO represents a particularly detrimental phenotype, combining the pro-inflammatory effects of obesity with the immunosuppressive and catabolic consequences of muscle depletion [59].

Other studies focused on BC variations between baseline and follow-up CT scans and their potential impact: in a comprehensive multicohort analysis conducted on more than 1700 patients, a subcutaneous adipose tissue (SAT) density increase of more than 5% and a loss in SMA greater than 5% were negatively associated with OS [60].

Moreover, BC features seem to impact both aspects of immunotherapy including adverse events: previous literature suggested that sarcopenia and low muscle attenuation (MA) were significantly associated with high-grade immune-related adverse events (irAEs) (OR 5.34, *p* = 0.033; OR 5.23, *p* = 0.013, respectively) [44]. Of note, recent studies showed that sarcopenic overweight was specifically linked to early acute limiting toxicity in patients with melanoma receiving anti-PD1 checkpoint inhibitors [61].

Although most studies have relied on single slice L3 computed tomography segmentation, volumetric approaches are emerging and may improve precision; moreover, skeletal muscle index cut off values differ by sex and likely by population and ethnicity, highlighting the need for consensus definitions and standardized measurement and reporting.

Differences in study design, populations, and measurements can yield divergent associations between BMI, BC, and immunotherapy outcomes. Key sources of heterogeneity likely include smoking history and intensity; performance status and comorbidities; cachexia, weight loss trajectory, and systemic inflammation; treatment line and regimen; tumor PD-L1 level and tumour mutational burden; definitions and cutoffs for skeletal muscle index and skeletal muscle density by sex and region; imaging approach single slice L3 versus volumetric segmentation; and analytic choices including immortal time bias, adjustment sets, and handling of missing data.

## Lipid Metabolism

### Lipid Metabolism as Critical Player of Anti-tumor Immune Responses

Lipid metabolism is a broad and multifaceted process that plays a crucial role in maintaining physiological balance and is intricately linked to both health and disease. Lipid metabolism encompasses the processes of absorption, transport, synthesis, catabolism, and utilization of lipids by the body [62, 63]. In fact, lipids serve as a vital source of energy for the body and are also a component of cell membranes, hormones, signalling molecules, and other essential biological molecules. Aberrant lipid metabolism has been associated with various disorders, including obesity, metabolic syndrome, and cardiovascular diseases [64, 65]. Moreover, its role in cancer is well established, as abnormal lipid metabolism is a main metabolic alteration, enabling tumor cells to generate energy, build cellular membranes, and promote proliferation, invasion, and metastasis. Intriguingly, lipid metabolism plays also a crucial role in immune system activity [66, 67]. However, most signals linking lipid profiles to outcomes on ICIs are observational and prone to confounding by general fitness, diet, and comorbidity. Higher total cholesterol or favorable lipoprotein patterns may correlate with better survival, but caution is needed in attributing causality.

### Cholesterol Metabolism and Cholesterol-modifying Drugs

When we talk about lipid metabolism, cholesterol often comes to mind. Cholesterol metabolism in the human body is a highly intricate process, maintained by a dynamic balance of synthesis, absorption, transport, and storage. Cholesterol is synthesized de novo through the mevalonate pathway, regulated by sterol regulatory-element binding proteins (SREBPs), or acquired via receptor-mediated uptake of LDL and HDL. Cellular cholesterol homeostasis is further maintained by several efflux mechanisms, including ABCA1 and ABCG1 transporters [67–69]. In recent years, increasing evidence has highlighted the impact of cholesterol metabolism on the efficacy of ICIs in cancer, supported by robust preclinical data and clinical observations. In fact, cholesterol plays a crucial role not only in supporting cancer cell survival, proliferation, and metastasis but also in regulating immune cell function [66].

The complexity relies on the fact that each of the regulators of cholesterol metabolism influence immune cell function in a context-dependent manner. In Tregs, SREBPs sustains immunosuppressive activity, and its inhibition enhances anti-tumor immunity [70]. Conversely, in natural killer (NK) cells, SREBPs are essential for metabolic reprogramming and cytotoxic activity, while in dendritic cells (DCs), SREBP2 activation suppresses antigen presentation, facilitating tumor immune evasion [71, 72]. ABCA1 and ABCG1 regulate cholesterol efflux and influence immune responses: ABCA1 deficiency impairs T cell receptor signalling, while ABCG1 loss promotes an anti-tumor M1 macrophage phenotype [73, 74]. Interestingly, intracellular cholesterol also modulates immune signalling: intracellular cholesterol depletion enhances dendritic cell maturation, stimulating a more efficient antigen presentation [75]. Moreover, in the tumor microenvironment, high cholesterol promotes CD8^+^ T cell exhaustion, increasing immune checkpoint expression and reducing cytotoxic function. Lowering cholesterol can restore T cell activity and enhances anti-tumor immunity [76]. Also, drugs that regulate cholesterol metabolism are used in clinical practice to renormalized blood cholesterol levels seems to impact biologically on anti-cancer immunity [77]. Statins, targeting HMG-CoA reductase (HMGCR), the rate-limiting enzyme in the mevalonate pathway, widely used for cardiovascular diseases, have demonstrated anti-tumor effects across multiple cancers.

By modulating vesicle trafficking and antigen presentation, statins enhance MHC/peptide complex stability, prolong antigen retention on DCs, and promote CD8^*+*^ T-cell activation [78]. In preclinical models, statins combined with ICIs significantly reduced tumor burden [79].

Circulating cholesterol is emerging as a potential biomarker influencing outcomes in cancer patients treated with ICIs. Several studies have been observed in broader cohorts, including melanoma, RCC, NSCLC, and urothelial carcinoma, where circulating higher total cholesterol but also higher HDL cholesterol levels were linked to longer survival under ICI-based treatment [80]. Moreover, blood cholesterol levels seem to be associated with circulating IL-6 levels, with patients with hypercholesterolemia showed lower IL-6, IL-10 and TNF-α blood concentrations [81]. while cholesterol levels seem to be associated with clinical outcomes to ICI independently of systemic inflammation as assessed by the neutrophil-to-lymphocyte ratio (NLR) [82].

Our group further developed a “LIPID score” integrating total cholesterol and triglyceride levels, effectively stratifying patients into good, intermediate, and poor survival categories [83]. Moreover, cholesterol homeostasis markers, including passive diffusion and efflux transporters ABCA1/ABCG1, correlated with prolonged survival, supporting the role of lipid metabolism in modulating the immune response [84].

Patients with cancer often take various non-cancer medications that can influence treatment outcomes through pharmacodynamic and pharmacokinetic interactions. Among these, statins, widely used for blood cholesterol levels regulation, have shown potential immunomodulatory effects that may enhance responses to ICIs. Our previous study showed that baseline statin use, specifically high intensity statins, improved outcomes in patients with advanced malignant pleural mesothelioma and NSCLC treated with PD-1 inhibitors [85]. However, evidence remains inconsistent across studies [86–88]. Other evidence in NSCLC and urothelial carcinoma found no correlation between statin use and survival, but lacked information regarding statin intensity [86–88]. Lipophilicity may also influence outcomes, as lipophilic statins have been linked to lower mortality in ICI-treated patients. Despite promising preclinical evidence, most clinical data are retrospective and subject to selection biases. Factors such as statin type, timing, and patient lipid profiles must be further explored. Prospective trials and preclinical models are needed to clarify whether statins, or other cholesterol-lowering agents like PCSK9 inhibitors, can enhance ICI efficacy and serve as viable adjuvant in cancer treatment.

It may seem contradictory at first that high cholesterol is associated with a better prognosis under ICIs, while statins, which reduce cholesterol levels, are known to enhance ICI efficacy. Beyond cholesterol levels, the quality of circulating lipoproteins may play a crucial role. The balance between HDL and LDL, rather than total cholesterol alone, appears to influence immune function. Statins’ broader effects, including their ability to reduce systemic inflammation and biomarkers associated with poor ICI response (such as IL-6 and C-reactive protein [CRP]), further complicate the picture. Additionally, statins improve cardiovascular health, which may indirectly enhance overall survival in cancer patients [89–92].

Therefore, it’s important to consider that statins alter the cholesterol profile by increasing HDL, which appears to enhance T cell anti-tumor activity and exert antioxidant effects, while simultaneously lowering LDL, which is linked to higher oxidative stress. Moreover, statins help reduce systemic inflammation and improve cardiovascular health, ultimately benefiting overall patient well-being [93, 94]. Therefore, statins may enhance ICI effectiveness not only by modulating cholesterol levels, antigen presentation, but also by addressing broader health factors that contribute to treatment outcomes.

Further studies should also evaluate the intensity and timing of statin use, blood cholesterol composition and the timing of dyslipidemia onset but also tumor tissue cholesterol content, and individual patient characteristics and comorbidities. This will help to more thoroughly understand how these factors influence the role of statins in enhancing ICI efficacy.

In summary, cholesterol metabolism plays a multifaceted role in cancer and immune regulation. While statins and other cholesterol-lowering agents hold promise as potential adjuncts to immunotherapy, their effects are likely mediated by multiple factors beyond cholesterol reduction alone. A deeper understanding of cholesterol’s influence on immune function, systemic inflammation, and tumor biology is needed to optimize therapeutic strategies and fully harness its potential in improving cancer treatment outcomes.

### Fatty Acid Metabolism

Beyond cholesterol, also fatty acids (FA) play a key role in cancer progression and immune cells function, providing energy to support cell growth, contributing to cell movement as essential components of membrane structure, and regulating signal transduction as key signalling molecules. Moreover, to not forget is the fact that SREBP-1c, the predominant SREBP-1 isoform in adult liver, preferentially activates genes required for FA synthesis, being a link between cholesterol and FA metabolism [95].

As described for cholesterol, also FA metabolism [96] plays a different functional role across immune cell types and is context-related. Previous findings showed that increased levels of polyunsaturated FAs (PUFAs) within the cell membrane can improve the efficacy of non-small cell lung cancer (NSCLC) to anti-PD-1 and anti-PD-L1 immunotherapy. By altering the lipid composition of the tumor cell membrane, increased PUFA incorporation may led to greater membrane fluidity and reduced binding between PD-L1 and PD-1, enhancing the efficacy of pembrolizumab in humanized NSCLC immune-xenografts [97]. Other FA, such as linoleic acid (LA), can boost CD8^+^ T cell activity in the TME: LA promotes Mitochondria-endoplasmic reticulum contacts (MERCs) [98], enhancing mitochondrial calcium signalling, mitochondrial function, and therefore CD8^+^ T cell anti-tumor activity both in vitro and in vivo. Other studies have demonstrated the role of FA metabolism in promoting tumor-associated macrophages (TAMs) in the TME [99]: TAMs internalize FA primarily through key lipid transporters, including FA translocase (CD36). High CD36 expression in tumors has been strongly associated with M2-like TAM infiltration and increased lipid accumulation, contributing to an immunosuppressive TME. Specifically, TAMs take up tumor-derived monounsaturated long-chain FA via CD36, promoting their polarization toward the M2 phenotype. In addition, recent studies have shown that lipid-rich TAMs exhibit diminished phagocytic activity and increased PD-L1 expression [100], further dampening T cell-mediated immunity and supporting tumor progression.

Clinical studies also demonstrated a prognostic role of circulating FA in patients treated with ICI: a recent study [101] showed that among 102 NSCLC patients, higher PUFA and monosaturated FA intake was significantly associated with longer clinical outcomes and were linked to a greater abundance of beneficial immunogenic bacteria, including Eubacterium, Alistipes, and Bifidobacterium, highlighting the potential role of dietary counseling as a strategy to modulate the and enhance immunotherapy efficacy. Another study [102] showed that, in a cohort of 112 NSCLC patients receiving immunotherapy, higher circulating levels of specific FA were positively correlated with improved clinical outcomes, suggesting again that the balance of these FA may influence membrane fluidity and receptor activity, potentially affecting ICI efficacy.

In conclusion, FA metabolism plays a pivotal role in cancer progression and immune function, significantly impacting the efficacy of ICI immunotherapy. A deeper understanding of the underlying biology at the single-cell level can provide valuable insights, facilitating the development of targeted therapies aimed at enhancing ICI efficacy. Moreover, clinical studies suggest that FA levels can predict treatment outcomes, highlighting the potential of dietary interventions to improve clinical outcomes under ICI [101].

## Glucose Metabolism

### Hyperglycemia, Diabetes, and Cancer Progression

Type 2 diabetes mellitus (T2DM) is highly prevalent among patients with cancer, with approximately 18% having T2DM at diagnosis and around 20% developing it subsequently, significantly impacting prognosis, treatment efficacy, and quality of life [103–105].

Chronic hyperglycemia, characteristic of diabetes mellitus (DM), has been increasingly recognized as a detrimental prognostic factor across several malignancies [106, 107], including NSCLC [108], due to its ability to sustain chronic systemic inflammation, oxidative stress, and altered immune functions. Elevated glucose levels directly enhance tumor growth by supplying energy and biosynthetic precursors critical for proliferation and metastatic dissemination. Moreover, persistent hyperglycemia fosters a pro-inflammatory microenvironment characterised by increased circulating levels of cytokines such as IL-1β, IL-6, and TNF-α, potentially facilitating cancer progression by promoting angiogenesis, invasion, and resistance to apoptosis [109, 110].

### Metabolic Reprogramming and T-cell Dysfunction Under Hyperglycemia

The chronic inflammatory milieu, typical of T2DM, can significantly contribute to impaired immune surveillance, enhancing the establishment of an immunosuppressive TME. Specifically, chronic hyperglycemia induces functional exhaustion of cytotoxic CD8^+^ T cells, which exhibit reduced proliferation, cytotoxic potential, and impaired production of key cytokines, such as IFN-γ and TNF-α, crucial for effective antitumor responses [111].

Chronic hyperglycemia can also promotes the formation of advanced glycation end-products (AGEs), generating reactive oxygen species (ROS) and engaging the polyol pathway, resulting in diminished glutathione (GSH) levels and related enzyme activity. This pathway disruption significantly alters cytokine profiles and immune responses, promoting an immunosuppressive TME and impairing innate and adaptive immune functions [112].

Glucose metabolism critically governs T-cell activation, differentiation, and effector function. Under physiological conditions, activated effector T cells rely on aerobic glycolysis to support rapid proliferation and effector cytokine secretion. However, chronic hyperglycemia in diabetic individuals induces metabolic dysregulation characterised by sustained activation of glycolytic pathways, leading paradoxically to T-cell exhaustion through continuous overstimulation. Exhausted T cells display increased expression of inhibitory checkpoint molecules such as PD-1, CTLA-4, and TIM-3, which correlate with impaired antitumor immunity and reduced efficacy of ICIs [111].

In addition, chronic systemic inflammation due to macrophage infiltration and Th1 and Th17 T-cell activation contributes significantly to a pro-inflammatory milieu typical of T2DM, further compromising immune surveillance and functionality [113]. Importantly, Tregs, known for their metabolic flexibility, thrive under high-glucose conditions, enhancing their suppressive functions. Enhanced glycolytic flux within Tregs promotes their proliferation and secretion of immunosuppressive cytokines such as IL-10 and TGF-β, exacerbating the immunosuppressive state within the TME. Importantly, targeting glycolytic pathways specifically in Tregs has shown potential in reversing their suppressive effects, thereby restoring effector T-cell responses and potentially enhancing the efficacy of ICIs in hyperglycemic conditions [114].

### Clinical Implications oOf Glycemic Control and Diabetes Management

Diabetes, particularly T2DM, has been consistently associated with worse outcomes across a wide range of tumor types. In addition to increasing the risk of cancer incidence, particularly for breast, colorectal, endometrial, liver, and pancreatic malignancies, diabetes also correlates with higher cancer-related mortality [105]. The negative prognostic impact of diabetes is believed to arise from a combination of factors, including hyperinsulinemia, chronic hyperglycemia, systemic inflammation, and immune dysfunction [105]. Poor glycemic control further exacerbates these effects, contributing to reduced treatment tolerance, higher infection rates, and shorter survival, particularly in patients with early-stage disease or long-term therapeutic needs.

In the context of ICI-based treatments, clinical evidence corroborates the potential detrimental impact of diabetes and poor glycemic control on the outcomes of cancer patients treated with ICIs. In a recent large-scale study involving 1,395 patients with solid tumors treated with ICIs, pre-existing T2DM was associated with poorer efficacy outcomes, accompanied by downregulated gene signatures related to adaptive and innate immune responses in tumor tissues. Furthermore, a linear relationship was observed between increased median blood glucose levels and elevated NLR, underscoring the role of systemic inflammation in mediating the negative impact of hyperglycemia on immune responses [115]. Importantly, elevated glycated hemoglobin (HbA1c) levels, reflecting long-term poor glycemic control, have emerged as a reliable biomarker predicting poorer response to immunotherapy, suggesting that rigorous management of glucose metabolism is critical to optimizing therapeutic outcomes in these patients [115–117].

Furthermore, there seems to be data regarding the potential different outcomes in modulating immunotherapy depending on the choice of antidiabetic medication, with data regarding metformin showing a possible context-specific role of this drug. In the above mentioned large multicentre retrospective analysis, Cortellini et al. [115] found that patients with T2DM receiving metformin at the start of ICIs had shorter PFS and OS than both patients with diabetes on other glucose lowering medications (GLMs) and non-diabetic controls, suggesting a deleterious, drug-specific immunomodulatory effect that cannot be explained by glycaemic control alone. Conversely, a recent translational study in early-stage NSCLC demonstrated an “obesity-specific” benefit: metformin improved relapse-free and overall survival and potentiated anti-PD-1 activity exclusively in overweight/obese patients, with no advantage in normal-weight individuals [118]. These apparently divergent findings underscore that metformin’s immunometabolic effects depend on the host’s nutritional and inflammatory milieu [119]. Moreover, in a preclinical study on syngeneic mouse colon and melanoma models, Zhan et al. explored the effect of specific GLMs with regards to tumor growth under anti-PD1 inhibition. They found that insulin and sulfonylureas may negatively affect immunotherapy responses by exacerbating hyperinsulinemia-induced immunosuppression, while drugs such as acarbose and sitagliptin, which specifically target postprandial hyperglycemia, show promise in enhancing antitumor immunity when combined with ICIs, suggesting that pharmacological strategies aimed at precise metabolic regulation can significantly impact cancer prognosis in diabetic patients [120]. Similarly, emerging data regarding GLP-1 agonists also suggest a possible role of these drugs in preventing disease progression and enhancing antitumoral response in patients with hepatocellular carcinoma (HCC) by enhancing NK cells antitumoral effect and reversing obesity-induced immune-impairment [121].

Collectively, these findings underscore the necessity of integrated metabolic and immunological strategies for the management of diabetic cancer patients, advocating for personalized therapeutic approaches that account for individual metabolic profiles. While evidence supports a potential link between T2DM and poorer outcomes ICIs [115], the underlying biological mechanisms remain incompletely understood. Notably, T2DM is frequently associated with increased BMI and weight gain, as also observed in real-world immunotherapy cohorts [115], raising important questions about how this aligns with the mentioned obesity paradox. This apparent contradiction highlights the complexity of the immunometabolic landscape in which a patient with T2DM is embedded.

Rather than representing a static condition, diabetes reflects the culmination of a decades-long trajectory of metabolic dysregulation. Along this continuum, individuals may transition through distinct immunological states. In early phases, modest adiposity, particularly subcutaneous fat accumulation, might support an immune-permissive environment, potentially enhancing responsiveness to ICIs. However, with progression toward insulin resistance, visceral and intermuscular fat accumulation, and chronic hyperglycemia, the immunometabolic milieu may become increasingly dysregulated. This evolution is often accompanied by low-grade inflammation, T cell exhaustion, myeloid dysfunction, and metabolic stress, ultimately leading to a decline in antitumor immunity and reduced efficacy of immunotherapy.

Such heterogeneity underscores the need for dynamic and individualized assessment of host metabolic status in cancer care, moving beyond BMI alone to capture the full complexity of adiposity, glycemic control, lipid metabolism and immune function across time.

## Sex Dimorphism and Hormonal Regulation

### Sex-specific Differences in Immunotherapy Efficacy

Sex, referring to the biological differences between men and women, influences the development and function of the immune system by interacting with genetic, biological, and environmental factors.

The role of these sex-based immune differences in shaping anticancer immune responses and immune evasion, and their impact on the efficacy of ICIs, is currently the focus of extensive research. However, in NSCLC, human evidence linking estrogen and androgen receptor signalling to immunotherapy outcomes is limited and sometimes indirect; we therefore present these mechanisms as hypotheses that require prospective testing.

A large comprehensive meta-analysis of randomized clinical trials conducted by Conforti et al. [122], demonstrated that male patients derive a greater survival benefit from ICIs compared to females across multiple advanced cancer types, including lung cancer. A subsequent analysis focusing specifically on advanced NSCLC revealed that women appear to benefit more from a combination of chemotherapy and ICIs compared to men [123]. Further supporting evidence comes from a pan-cancer meta-analysis of over 1,000 patients treated with ICIs, where a machine-learning model integrating genomic, immune, and clinical variables identified male sex as a significant positive predictor of response [124].

To investigate the determinants of these findings, it is important to focus on how sex dimorphism influences the quantity and functionality of key effector and suppressor cells within the TME.

A recent comprehensive transcriptomic and molecular analysis of NSCLC tumors [125] revealed that women exhibit a stronger and more structured immune response against the tumor, with higher immune cell infiltration in the TME. However, this heightened immune activity is accompanied by the onset of complex resistance mechanisms, with increased expression of inhibitory immune-checkpoint molecules and a greater presence of immunosuppressive cells. In contrast, men tend to display a more immune-excluded phenotype, with impaired antigen presentation and a higher frequency of human leukocyte antigen (HLA) loss of heterozygosity. As a clinical implication of these findings, tumor mutational burden (TMB) and survival outcomes correlates different between sexes: a linear association was observed in women, whereas in men, a survival benefit emerged only at high TMB thresholds, suggesting the need for sex-specific TMB cut-offs to optimize ICI response.

In the contest of these TME sex-based differences, sex hormones play a central role and can act as a modulator of response to immunotherapy.

Androgen receptor (AR) signalling has been associated with an exhausted T-cell phenotype and downregulation of key immune mediators, contributing to a dysfunctional antitumor response [126]. Supporting this, in patients with advanced prostate cancer, AR inhibition combined with PD-1 blockade has shown improved responses, likely due to their synergistic effect in preventing T-cell exhaustion and enhancing cytotoxic activity [127, 128].

In a preclinical study on murine melanoma models, estrogen signalling through estrogen receptor alpha (ERα) promotes the polarization of TAMs toward an immunosuppressive M2 phenotype, which impairs cytotoxic T-cell function and limits ICI efficacy. Further, this study demonstrated that inhibiting estrogen signalling with the selective estrogen receptor downregulator (SERD) fulvestrant reduces tumor growth by enhancing the presence of activated CD8^+^ T cells in the TME, improving the efficacy of ICIs [129].

These findings underscore the need for further investigation into therapeutic strategies integrating ICIs with hormonal modulation, highlighting their potential as a targeted approach to reverse immune suppression and enhance ICI efficacy in both male and female patients.

### Hormonal Regulation of Adiposity and Immunity

The distribution of AT differs significantly between males and females, shaping distinct metabolic and immune environments. Males predominantly accumulate visceral AT (VAT), whereas in females, subcutaneous AT (SAT) is the primary storage site during the premenopausal phase, while VAT becomes more prominent after menopause. These sex-related differences in AT distribution may have significant implications for immune response and balance, particularly playing a crucial role in long-term immunological memory and host defence [130].

As previously described, obesity has been increasingly recognized as a factor influencing immune function, with several study highlight its association with improved survival and response to ICIs [27, 28, 55, 131]. Consistent with our body composition section, fat distribution is a plausible mediator between sex hormones, inflammaging, and T cell exhaustion, with visceral versus subcutaneous adiposity representing candidate pathways to be evaluated prospectively.

In a retrospective study [55] on patients with unresectable or metastatic melanoma treated with ICIs, increased VAT correlated with longer OS, although systemic inflammation was identified as a potential confounder. Notably, no significant sex-based differences in clinical outcomes were observed in this cohort.

In contrast, McQuade [132] et al. observed that in pts with metastatic melanoma obesity was associated with improved survival for immunotherapy limited to male patients, with subsequent studies corroborated this sex-specific association [49, 133], suggesting that sex-linked differences in obesity may play a role regarding the “obesity paradox” in cancer immunotherapy.

Although the mechanisms underlying this phenomenon remain poorly understood, sex hormone regulation likely plays a key role in the complex interplay between sex, adiposity, and immune modulation.

In male, obesity is associated with reduced testosterone levels and moderate increases in estrogen, which may create a hormonal balance favouring ICI response​. In contrast, female obesity often results in higher estrogen levels, which have been linked to immunosuppressive effects, including M2 macrophage polarization and impaired CD8^+^ T cell effector function [18].

Preclinical models further support this hormonal influence. Recent evidence shown that in tumor-bearing, diet-induced obesity mouse models, tumor progression was greater in both male and female compared to control diet recipients. However, anti-PD(L)−1 as monotherapy was significantly effective in obese male mice, whereas obese females and lean mice of both sexes did not experience the same benefit. Interestingly, ovariectomy in female mice led to both greater weight gain and improved response to immunotherapy, comparable to that observed in obese males. Clinical data in melanoma patients mirrored these findings, as improved survival and treatment efficacy were observed only in high-BMI male patients [134].

In conclusion, although hormonal balance may contribute to the improved response to ICIs observed in obese male patients, further research is needed to elucidate the interplay between sex, fat distribution, metabolic status, and immunotherapy outcomes. Unravelling these mechanisms may shed light on sex-specific determinants of immune response and support the development of more tailored therapeutic strategies.

### Aging and Hormonal Changes

Aging and reproductive status of patients are associated with changes in sex hormone levels, body composition, and immune function, all of which can influence responses to ICIs.

Notably, the immunomodulatory effects of female hormones are highly context-dependent. During pregnancy, women develop tolerance to paternal-fetal antigens, driven by systemic expansion of regulatory T cells and other mechanisms that persist after delivery [135]. A unique consequence is fetal microchimerism (FMC), the long-term persistence of fetal cells in maternal blood and tissues. These cells, which can differentiate into immune or endothelial lineages [136], may influence maternal immune surveillance, tumor biology, and potentially affect antitumor immunity under ICI therapy [137]. Although current evidence remains largely descriptive, FMC effects suggest that previous pregnancies could represent an underexplored host factor shaping immune responses in cancer patients [137].

Conversely, the progressive decline in estrogen and testosterone levels observed during aging disrupts immune homeostasis, leading to increased systemic inflammation and immunosenescence. Indeed, aging is characterised by increased secretion of pro-inflammatory cytokines, such as TNF-α, IL-6, and IL-1β [126, 138, 139], along with a decline in naïve T cells and an increase in monocyte and cytotoxic cell functions [140], contributing to a chronic inflammatory state that impairs immune surveillance and response to immunotherapy.

Another consequence of aging is the progressive modification of muscle mass, which can significantly influence immune function and response to ICIs.

Interestingly, recent evidence from metastatic RCC suggests that muscle composition assessment may influence ICI efficacy depending on the treatment combination. In this study, myosteatosis was associated with poorer OS and PFS in patients treated with PD-1 inhibitor plus tyrosine kinase inhibitors, but showed a protective effect on PFS in those receiving PD-1 plus CTLA-4 blockade, potentially due to differences in immune cell composition and checkpoint expression within the TME [141].

Among these aging-related muscle modifications, sarcopenia has been identified as an independent negative prognostic factor and a predictor of poor response to ICIs [142]. Sarcopenia is associated with chronic low-grade inflammation and impaired T-cell function, both hallmark features of an aging immune system [143]. Elevated systemic inflammation often results in peripheral blood neutrophilia and reactive lymphopenia, leading to an increased NLR [144].

In advanced NSCLC patients treated with ICIs, a higher NLR has been consistently associated with poorer survival outcomes [144], supporting its role as a biomarker of immune dysfunction. Notably, an increased NLR has also been correlated with an immune-exhausted TME, characterised by decreased immune cells infiltrations [145].

A recent retrospective study investigating age-related immunophenotypic correlates in older adult with NSCLC receiving ICIs confirmed that this population exhibit a distinct TME compared to younger patients, enriched with multiple immunosuppressive checkpoints [146]. However, older adults remain underrepresented in clinical trials investigating ICI efficacy, leading to a gap in knowledge regarding optimal treatment strategies for this subgroup of patients.

Taken together, these considerations raise important questions about the role of aging and sex-related differences as key parameters in predicting and optimizing responses to cancer immunotherapy, warranting further investigation and greater inclusion of older adults in clinical trials to ensure tailored treatment strategies for this underrepresented population.

## Space for Intervention

Consistent evidence links host energy metabolism to variability in response to immunotherapy in NSCLC, yet most signals remain observational and may be confounded by general fitness, comorbidity, treatment line, and disease biology.

Nevertheless, a pragmatic and biologically grounded space for intervention exists while interventional trials mature: avoid persistent hyperglycemia with objective monitoring of fasting glucose and HbA1c and individualised glucose lowering therapy; treat dyslipidemia according to cardiovascular prevention standards rather than with the sole intent of enhancing immunotherapy; and identify low muscle mass and myosteatosis early with computed tomography based metrics using sex and population appropriate cutoffs, triggering nutrition and structured exercise programmes with supportive care referral as appropriate.

In line with the modifiable host factor framework articulated by Betof Warner and McQuade [147] and colleagues, these measures prioritise risk reduction and standardisation rather than unproven claims of immunotherapy specific benefit. However, a growing body of evidence links physical exercise and immunotherapy, suggesting that structured aerobic and resistance training exerts measurable systemic immunomodulation through leukocyte mobilisation, improved T cell competence and myokine signalling, and should be prospectively tested as a co intervention with predefined dose and objective fitness endpoints [148].

Dietary interventions beyond conventional regimens for metabolic and cardiovascular prevention, including fasting mimicking protocols, are also under consideration. Where appropriate and under clinical supervision, short periodic fasting mimicking diet cycles, as explored by Vernieri and colleagues, can be evaluated within structured programmes given their reproducible effects on glucose, insulin, IGF-1 and immune signatures [149].

In the context of glycemic control, improving glucose homeostasis by any effective means enhances cardiometabolic health and reduces systemic inflammatory burden, variables associated with a lower overall risk of disease progression, even though immunotherapy specific benefits remain to be established [105]. In this scenario, drug class may matter for both biology and tolerability: signals for metformin vary by metabolic phenotype and may reflect improved glycemic control and weight rather than a direct anticancer effect [115, 118, 119]; insulin and sulfonylureas often mark more advanced or hyperinsulinemic disease and may associate with poorer outcomes through confounding by indication; SGLT2 inhibitors and GLP-1 receptor agonists improve glycaemia and weight but immunotherapy specific signals are preliminary; evidence for DPP-4 inhibitors and acarbose is early and heterogeneous [105, 120, 121].

In the immunotherapy setting, lipid control should follow established cardiovascular and metabolic guidelines rather than pursue immunotherapy specific goals [150, 151]. Practically, this means prioritising lifestyle measures and using statins as first line therapy for hypercholesterolemia, with high intensity statin indicated for clinical atherosclerotic disease and ezetimibe/Proprotein Convertase Subtilisin/Kexin type 9 (PCSK9) inhibitors if clinical targets are still unmet, while fibrates and omega 3 fatty acids can be prescribed for the treatment of hypertriglyceridemia.

To move the field forward, it is essential to standardise phenotyping at baseline and during treatment and to harmonise reporting of metabolic markers, body composition measures, and concomitant medications. However, these steps are not substitutes for randomised evidence, but they can potentially reduce reversible metabolic stress, standardise supportive care, and generate testable hypotheses for immunotherapy specific benefit; they are summarised in the accompanying decision aid Table 3.

**Table 3:** Host-related factors as potential biomarkers and intervention targets in patients treated with immune checkpoint inhibitors (ICIs)

Host factor	How to measure	Association with IO outcomes	Candidate intervention	Strength of evidence	Practical note
Muscle mass and myosteatosis	CT derived skeletal muscle index and density; simple weight loss screen	Consistently adverse	Nutrition plus exercise programmes; early supportive care	Moderate for association, low for intervention impact	Reassess during treatment
Body mass index and fat distribution	BMI; CT subcutaneous and visceral fat indices	Mixed for BMI, nuanced by depot	Lifestyle counselling	Moderate for association	Interpret BMI with body composition
Dyslipidaemia	Lipid panel including HDL, LDL, triglycerides	Signals for favourable lipoprotein patterns	Statins per guidelines; consider ezetimibe, PCSK9, fibrates where indicated	Moderate for association, low for IO specific benefit	Document statin class and intensity
Type 2 diabetes and hyperglycaemia	Fasting glucose, HbA1c	Adverse signals	Optimise glycaemic control; metformin where appropriate	Moderate for association	Avoid glucotoxicity
Sex hormones and adiposity	Clinical sex; adiposity distribution	Hypothesis generating	None specific; individualise	Low	Research priority
Ghrelin axis and cachexia	Weight loss; appetite	Mechanistic link to metastasis	Ghrelin agonists for appetite and weight endpoints	Low for IO outcomes	Supportive care focus

*CT* computed tomography; *IO* immunotherapy; *BMI* body-mass index; *HDL* high density lipoprotein; *LDL* low density lipoprotein; *PCSK*9 proprotein convertase subtilisin/Kexin type 9; *HBA1c* glycated emoglobin

## Conclusion

Response to ICIs in NSCLC is not solely determined by tumor-intrinsic factors but is profoundly shaped by a complex and dynamic network of host-related variables. Body composition, metabolic health, systemic inflammation, cholesterol homeostasis, sex-based immunobiology, and comorbidities such as type 2 diabetes mellitus collectively contribute to shaping the tumor–immune ecosystem. Far from being static, these factors evolve across time, forming a continuum that may influence both the onset and trajectory of immune responsiveness. Moreover, even if this review deliberately centres on host energy metabolism and body composition, the complexity of host determinants extends beyond what we synthesise here, encompassing the gut microbiome and its modifiers, including concomitant medications such as proton pump inhibitors and antibiotics.

This evolving host landscape challenges simplistic interpretations of phenomena like the “obesity paradox,” calling instead for integrative models that account for the temporal interplay between nutrition, metabolism, inflammation, and immune competence. If this complexity is properly understood, targeted strategies, including body composition optimization, glycemic control, and lipid modulation, could be leveraged to improve immunotherapy efficacy and patient prognosis. Future research should adopt a longitudinal, multi-parametric and/or artificial intelligence (AI) approaches, leveraging advanced imaging, omics technologies, and real-world data to refine patient stratification and personalize immunotherapy beyond tumor-based biomarkers. Understanding and addressing the host–tumor–immunity interface is essential to fully harness the potential of immunotherapy in NSCLC and transform it from a probabilistic intervention to a truly tailored strategy.

## Key References

- Vick L V., Rosario S, Riess JW, Canter RJ, Mukherjee S, Monjazeb AM, et al. Potential roles of sex-linked differences in obesity and cancer immunotherapy: revisiting the obesity paradox. npj Metabolic Health and Disease. 2024 May 23;2(1):5
  - Explores sex-linked differences in obesity and cancer immunotherapy, providing mechanistic insights into the obesity paradox.
- Nie W, Lu J, Qian J, Wang SY, Cheng L, Zheng L, et al. Obesity and survival in advanced non-small cell lung cancer patients treated with chemotherapy, immunotherapy, or chemoimmunotherapy: a multicenter cohort study. BMC Med. 2024 Oct 14;22(1):463.
  - Large multicenter cohort assessing the impact of BMI on survival in NSCLC patients receiving immunotherapy, challenging the generalizability of the obesity paradox.
- Lou J, Guo Y, Li L, Yang Y, Liu C, Zheng C, et al. Explanation of the obesity paradox of immunotherapy in cancer patients using CT-derived adipose composition parameters: A systematic review and meta-analysis. Int Immunopharmacol. 2025 Jan;144:113699.
  - Systematic review and meta-analysis linking CT-derived adipose composition with immunotherapy outcomes, refining the interpretation of BMI-based studies.
- Cortellini A, D’Alessio A, Cleary S, Buti S, Bersanelli M, Bordi P, et al. Type 2 Diabetes Mellitus and Efficacy Outcomes from Immune Checkpoint Blockade in Patients with Cancer. Clinical Cancer Research. 2023 Jul 14;29(14):2714–24.
  - Demonstrates the negative prognostic impact of type 2 diabetes and hyperglycemia in patients treated with ICIs, emphasizing host metabolic influence.

## Data Availability

No datasets were generated or analysed during the current study.
